# Therapy of Experimental NASH and Fibrosis with Galectin Inhibitors

**DOI:** 10.1371/journal.pone.0083481

**Published:** 2013-12-18

**Authors:** Peter G. Traber, Eliezer Zomer

**Affiliations:** 1 Galectin Therapeutics Inc, Norcross, Georgia, United States of America; 2 Department of Medicine, Emory University School of Medicine, Atlanta, Georgia, United States of America; University of Navarra School of Medicine and Center for Applied Medical Research (CIMA), Spain

## Abstract

Non-alcoholic steatohepatitis (NASH) and resultant liver fibrosis is a major health problem without effective therapy. Some data suggest that galectin-3 null mice are resistant to the development of NASH with fibrosis. We examined the ability of two complex carbohydrate drugs that bind galectin-3, GM-CT-01 and GR-MD-02, to treat NASH with fibrosis in a murine model. GR-MD-02 treatment resulted in marked improvement in liver histology with significant reduction in NASH activity and collagen deposition. Treatments seemed also to improve both glomerulopathy and interstitial fibrosis observed in kidneys. The improvement in liver histology was evident when animals were treated early in disease or after establishment of liver fibrosis. In all measures, GM-CT-01 had an intermediate effect between vehicle and GR-MD-02. Galectin-3 protein expression was increased in NASH with highest expression in macrophages surrounding lipid laden hepatocytes, and reduced following treatment with GR-MD-02, while the number of macrophages was unchanged. Treatment with GR-MD-02 also reduced the expression of pathological indicators including iNOS, an important TH1 inflammatory mediator, CD36, a scavenger receptor for lipoproteins on macrophages, and α-smooth muscle actin, a marker for activated stellate cells which are the primary collagen producing cells in liver fibrosis. We conclude that treatment with these galectin-3 targeting drugs improved histopathological findings of NASH and markedly reduced fibrosis in a murine model of NASH. While the mechanisms require further investigation, the treatment effect is associated with a reduction of galectin-3 expressed by activated macrophages which was associated with regression of NASH, including hepatocellular fat accumulation, hepatocyte ballooning, intra-portal and intra-lobular inflammatory infiltrate, and deposition of collagen. Similar effects were found with GM-CT-01, but with approximately four-fold lower potency than GR-MD-02. The results, in combination with previous experiments in toxin-induced fibrosis, suggest that these galectin-targeting drugs may have potential in human NASH with fibrosis.

## Introduction

Nonalcoholic fatty liver disease (NAFLD) and non-alcoholic steatohepatitis (NASH) are common liver disorders in the United States [1]. It is estimated that worldwide prevalence of NAFLD ranges from 6.3% to 33% with a median of 20% in the general population among multiple studies based on a variety of assessment methods [1]. In high risk groups of severe obesity, type-2 diabetes, and dyslipidemia, the prevalence of NAFLD was found to be 90%, 69% and 50%, respectively. A subset of individuals with NAFLD are found to have NASH, which is excessive fat accumulation in hepatocytes (steatosis) with the addition of inflammatory cell infiltrates, evidence of damage to hepatocytes (ballooning degeneration), and the deposition of fibrous tissue. It is estimated that between 3–5% of Americans are affected by NASH [1]. For patients in the early stages of NASH, about 33% will progress to advanced fibrosis (stage 3 and 4-cirrhosis) over 5–10 years [2]. Among those who develop NASH cirrhosis, 25% will develop major complications of portal hypertension within three years [2]. As a result, patients with NASH have increased overall mortality with an increased liver-related mortality [3], [4]. The only therapy currently available for these advanced patients is liver transplantation. The percentage of liver transplantations performed in the US for NASH is between 10 and 15%, but the numbers are increasing and it has been suggested that it may become the leading cause for liver transplantation over the next 20 years [5]. Currently, there are no FDA-approved medical therapies for NASH or liver fibrosis. There is an urgent need for new therapeutic approaches that are not only effective in ameliorating fat accumulation, cell death, and inflammation, but also is effective at reducing or reversing fibrosis.

Galectin-3 protein (gal-3), a member of a family of proteins which have the property of binding to terminal galactose residues in glycoproteins [6], has been implicated in the pathogenesis of liver fibrosis as well as in other organ fibrogenesis. Gal-3 null mice are resistant to liver fibrosis due to toxin administration [7], lung fibrosis due to bleomycin toxicity [8], and kidney fibrosis due to ureteral ligation [9]. Therefore, gal-3 appears to play a critical role in parenchymal fibrogenesis. We have previously reported that GR-MD-02 and GM-CT-01, gal-3 inhibitors are able to reverse fibrosis and cirrhosis in rats rendered cirrhotic by treatment with thioacetamide [10].

With respect to NASH, the effect of gal-3 on the pathological process has given mixed results in experiments using gal-3 null mice. Iacobini, et al. [11] have shown that in response to a high fat diet, normal mice readily developed fatty liver, inflammatory infiltrates, ballooning hepatocytes, and fibrosis, whereas the gal-3 null mice were resistant to the development of NASH and fibrosis. In contrast, Nomoto et al. found that gal-3 null mice at six months of age spontaneously developed pathological findings consistent with NASH [12] and at 15 months there was evidence of neoplastic nodule formation [13]. Moreover, using the choline-deficient L-amino-acid-defined (CDAA) diet model of NASH the same authors found that steatosis and cellular necrosis were greater in the gal-3 null mice than in wild-type mice [14]. Iacobini, et al. report following their gal-3 null mice for 24 months and did not find the effects reported by the other authors [11]. There is no obvious explanation for the different findings of these two groups.

In these studies, we used the same gal-3 inhibitors that showed a robust effect on thioacetamide-induced liver fibrosis in rats [10] to evaluate their effect in a murine model of NASH. Diabetic mice fed a high fat diet [15] were used to evaluate pharmacological inhibition of gal-3 using GR-MD-02 and GM-CT-01, two complex carbohydrate drugs that bind gal-3. Evaluation of the NASH model included histopathology evaluations of NASH, including hepatocellular fat accumulation, hepatocyte ballooning, intra-portal and intra-lobular inflammatory infiltrate, and deposition of collagen. In addition, the study evaluated the inflammatory mediator iNOS, the ALP/AGP scavenger receptor, and α-smooth muscle actin, a marker for activated stellate cells. Treatment with GR-MD-02 significantly improved NASH activity and markedly reduced fibrosis in this mouse model of NASH. Similar effects were found with GM-CT-01, but with approximately 4-fold lower potency than GR-MD-02. The results suggest that these galectin targeting drugs may have potential in human disease.

## Materials and Methods

### Drug Compounds

GM-CT-01 is a proprietary galactomannan polysaccharide comprised of predominantly mannose with single α-galactose side chains (mannose to galactose ratio of 1.7) with mean molecular weight of approximately 50 KDa, produced as described in patent US 7,893,252. GR-MD-02 is a proprietary galactoarabino-rhamnogalacturonan polysaccharide polymer that is comprised predominantly of galacturonic acid, with β-galactose and arabinose side chains of approximately 50 KDa that was produced as described in patent PCT/US12/55311.

### NASH mouse model

In these studies, we present the results of a series of experiments in a mouse model of NASH. The three experiments presented include the evaluation of twice weekly intravenous treatment starting at early and later times in the evolution of NASH, the evaluation of dose escalation of once weekly intravenous administration, and the evaluation of different routes of administration including intravenous, subcutaneous, and oral. C57BL/6J mice (14-day pregnant females) were obtained from CLEA-Japan (Tokyo, Japan) and NASH induced in male newborn mice by a single subcutaneous injection of streptozotocin (Sigma, St. Louis, MO) solution 2 days after birth and feeding with a high fat diet after four weeks of age, as previously described [15]. The caloric content of the high fat diet was comprised of 56.7% of calories from fat, 20.1% from proteins, and 23.2% from nitrogen free extract consisting of carbohydrates, sugars, starches, and hemicellulose which was provided to the mice ad libitum (cat# HFD32, CLEA-Japan, Japan). Vehicle and test substances at the doses indicated were administered intravenously in a volume corresponding to 5 mL/kg body weight. All animals used in the study were housed and cared for in accordance with the Japanese Pharmacological Society Guidelines for Animal Use. The animal study protocols used in this study were approved by animal use ethics committee at the Stelic Institute. Animals were fasted for 3 hours before sacrifice which was performed by exsanguination through direct cardiac puncture under ether anesthesia.

### Whole blood and plasma biochemistry

Fasting blood glucose was measured in whole blood samples using G Checker (Sanko Junyaku Co. Ltd., Japan). Plasma levels of AST, ALT, total bilirubin, creatinine, and TG were measured by FUJI DRY CHEM 7000 (Fuji Film, Japan).

### Liver biochemistry

To quantify liver hydroxyproline content, a quantitative assessment of collagen content, frozen liver samples (40–70 mg) were processed by an alkaline-acid hydrolysis method [15]. Protein concentrations of liver samples were determined using a BCA protein assay kit (Thermo Scientific, Rockford, IL) and used to normalize hydroxyproline content. Total liver lipid-extracts were obtained from caudate lobes by Folch's method [16] and liver TG levels were measured using the Triglyceride E-test (Wako, Japan) [15].

### Histopathological and immunohistochemical analyses

For H&E staining, sections were cut from paraffin blocks of liver tissue prefixed in Bouin's solution and stained with Lillie-Mayer's Hematoxylin (Muto Pure Chemicals, Japan) and eosin solution (Wako, Japan). To visualize collagen deposition, Bouin's fixed liver sections were stained using picro-Sirius red solution (Waldeck GmbH & Co. KG, Germany). NAFLD Activity score (NAS) was calculated according to previously described criteria [11], [17]. Scoring using NAS was performed a pathologist who was blinded to treatment group using the three criteria of steatosis, hepatocyte ballooning, and lobular inflammation. Steatosis in hepatocytes was scored as 0, 1, 2, or 3 if there were less than 5%, 5–33%, 33–66% and greater than 66% hepatocytes with fat, respectively. Hepatocyte ballooning was scored as 0 if there was none, 1 if there were few ballooned cells, and 3 if there were many cells with prominent ballooning. Lobular inflammation was scored as 0, 1, 2, or 3 if there were none, less than 2, 2–4, or greater than 4 inflammatory foci per 200× field, respectively. These individual scores were summed to give the NAS for each animal.

For immunohistochemistry, frozen sections were incubated with the optimal dilutions of rat anti-mouse F4/80 (cat# T2008, BMA), rabbit anti-mouse α-SMA (cat # 1184-1, Epitomics, Inc.), rat anti-mouse Gal-3 (cat # CL8942AP, Cedarlane), rabbit anti-mouse iNOS (cat # ab80978, abcam), or rabbit anti-mouse CD36 (ab15323, abcam) antibodies. Enzyme-substrate reactions were performed using DAB/H2O2 solution (Nichirei, Japan).

For quantitative analysis of fibrosis areas, bright field images of Sirius red-stained sections were captured using a digital camera (DFC280, Leica, Germany) around central veins at 200-fold magnification, and the positive areas in 36 fields (nine fields/section from four different sections) were measured using ImageJ software (National Institute of Health, Bethesda, MD). For quantitative analysis of immunohistochemistry, bright field images of immunostained sections were captured around central veins at 200-fold magnification, and the positive areas in five fields per section were measured.

### Quantitative PCR

Total RNA was extracted from liver samples using RNAiso (TaKaRa, Japan) according to the manufacturer's instructions. One µg of RNA was reverse-transcribed and real-time PCR was performed using Real-time PCR DICE and SYBR premix Taq (TaKaRa, Japan). PCR-primer sets were used for Gal-1 and Gal-3. The relative mRNA expression level normalized to Rplp0 (36B4) mRNA level.

- Lgals1 (gal-1)
  1. forward: 5′-TTCGCTTCAGCTTCAATCATGG-3′
  2. reverse: 5′-TGTTAGGCACAGGTTGTTGCTGTC-3′
- Lgals3 (gal-3)
  1. forward: 5′-CATTGTGTGTAACACGAAGCAGGAC-3′
  2. reverse: 5′-CTGCAGTAGGTGAGCATCGTTGA-3′
- Rplp0 (36B4)
  1. forward: 5′-TTCCAGGCTTTGGGCATCA-3′
  2. reverse: 5′-ATGTTCAGCATGTTCAGCAGTGTG-3′

### Statistical Analysis

In multiple groups experiments with parametric data, the differences between groups was assessed using one-way ANOVA to determine overall significance followed by Bonferroni Multiple Comparison Test or Tukey's Multiple Comparison Test to evaluate differences between individual groups. In situations when the data was not normally distributed because of outliers, the data were also analyzed using the Mann-Whitney U Test. Results are expressed as mean ± standard deviation and p values of <0.05 were considered significant.

## Results

### Treatment efficacy is independent of timing of intervention

Treatment of NASH mice with GM-CT-01 and GR-MD-02 was performed at two different time points to evaluate both the effect on NASH pathology and the dependency of that effect on the timing of intervention. Times for intervention with galectin inhibitors were chosen to coincide with the development of NASH (6 weeks after birth, early treatment cohort) and after the establishment of fibrosis (9 weeks after birth, late treatment cohort), as previously reported [15]. NASH mice in each treatment cohort (n = 6) were intravenously administered vehicle (0.9% sodium chloride), GM-CT-01 120 mg/kg, or GR-MD-02 60 mg/kg twice a week for 4 weeks. Body weight did not differ significantly between the vehicle group and either of the treatment groups. While three mice died (one in GM-CT-01 and two in vehicle); there were no abnormal necropsy findings except for the typical hepatic lesions of NASH. Deterioration in general condition was not observed in animals of any group during the experiment.

There were no significant differences in mean liver weight, liver-to-body weight, spleen weight, spleen-to-body weight ratio, blood glucose, bilirubin, creatinine, or triglyceride levels between the vehicle group and either of the treatment groups (data not shown). There was elevation of serum transaminase levels in the early cohort groups with levels of AST of 151 (SD 81) IU/L, 124 (SD 27) IU/L, and 140 (SD 57) IU/L and ALT levels of 44 (SD 15) IU/L, 47 (SD 9) IU/L, and 43 (SD 10) IU/L, for the vehicle, GM-CT-01 and GR-MD-02 groups, respectively. Similarly serum transaminase levels in the late cohort groups were levels of AST of 140 (SD 11) IU/L, 212 (SD 60) IU/L, and 144 (SD 76) IU/L and ALT levels of 50 (SD 12) IU/L, 65 (SD 34) IU/L, and 40 (SD 10) IU/L, for the vehicle, GM-CT-01 and GR-MD-02 groups, respectively. There were no significant differences in plasma AST or ALT levels between the vehicle group and either of the treatment groups in either early or late treatment cohorts.

**Figure 1:**
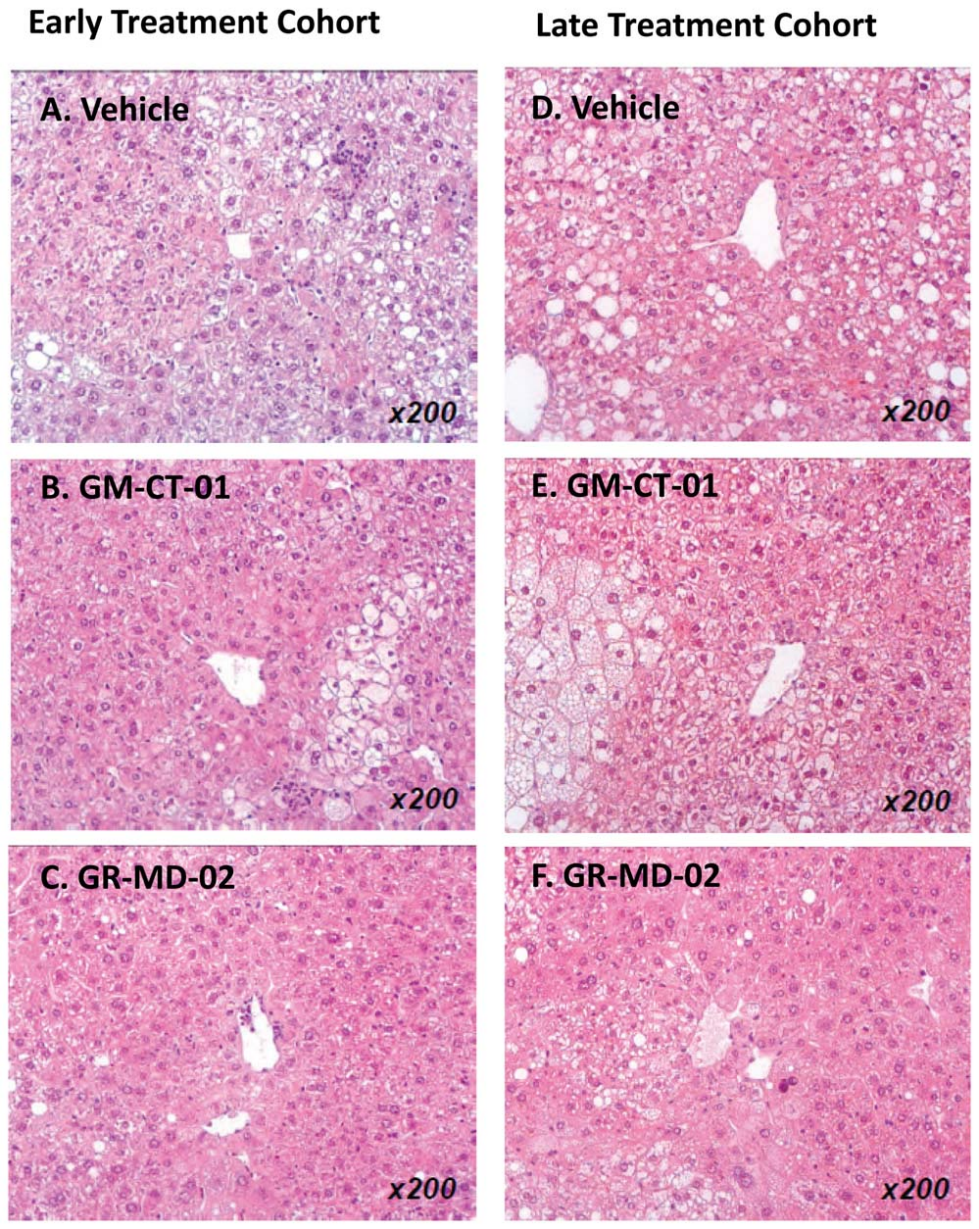
Liver histology stained with H&E. Early and late treatment cohorts were administered intravenously twice weekly vehicle (0.9% NaCl), GM-CT-01 (120 mg/kg), or GR-MD-02 (60 mg/kg). Images are representative of each group of animals as selected by the examining pathologist.

**Figure 2:**
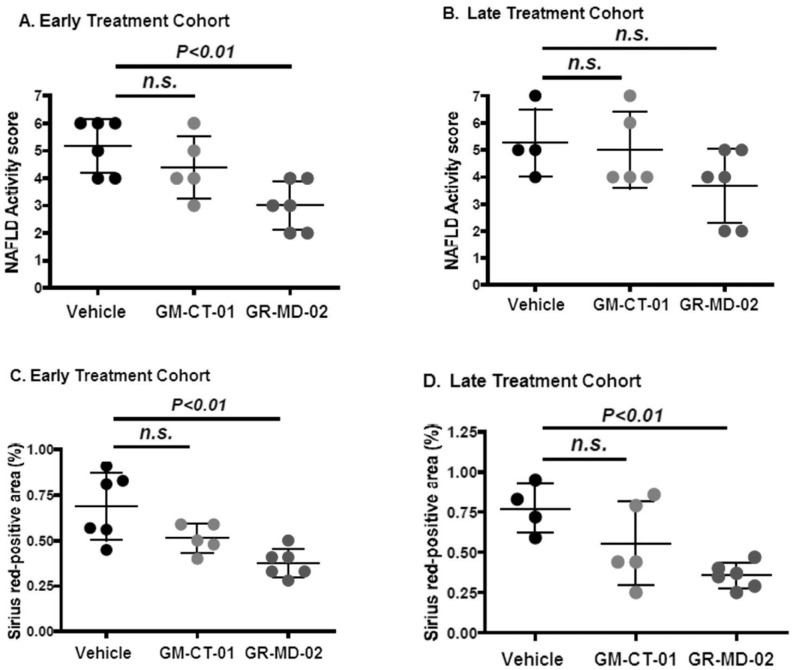
Quantification of NAFLD Activity Score (NAS) and collagen area. Figure 2A and 2B show NAFLD Activity Score for groups administered twice weekly intravenous injections of vehicle (0.9% NaCl), GM-CT-01 (120 mg/kg), or GR-MD-02 (60 mg/kg) for the early and late treatment cohorts, respectively. Figure 2C and 2D show percent Sirius red positive area for groups administered twice weekly intravenous injections of vehicle (0.9% NaCl), GM-CT-01 (120 mg/kg), or GR-MD-02 (60 mg/kg) for the early and late treatment cohorts, respectively. Differences between the vehicle group and the treatment groups were assessed by one way ANOVA followed by Bonferroni Multiple Comparison Test. *P* values <0.05 were considered significant and results expressed as mean ± SD.

Histological analysis of the liver showed an effect of treatment in both early and late treatment cohorts. Representative histological sections are shown in Figure 1 and the scoring results in Table 1.The vehicle groups showed micro-and macro-vesicular fat deposition, lobular inflammatory cell infiltrate, and hepatocellular ballooning which resulted in an average NAS of 5.2 and 5.3 for the early and late cohorts, respectively (Table 1). The GM-CT-01 groups had a tendency to decreased fat deposition and inflammation, but there were no changes in ballooning which resulted in an average NAS of 4.4 and 5.0 for the early and late cohorts, respectively (Table 1) These NAS were not significantly different than the vehicle group (Figures 2A and 2B). In the GR-MD-02 groups, the fat deposition, hepatocellular ballooning, and inflammatory infiltrate were decreased (Figures 1C and 1F) and consistent with these findings, the NAS was significantly improved (NAS score of 3.0) in the early treatment cohort compared with the vehicle group (Figures 2A and 2B). The late treatment cohort GR-MD-02 group showed improvement in the NAS versus vehicle control, although the value did not reach significance likely because of reduction in number of vehicle animals due to the death of two animals in the vehicle group. The degree of reduction in NAS in the GR-MD-02 treatment groups was of a similar magnitude to the reductions seen in gal-3 null mice that were fed a high fat diet, as described by Iacobini, et al. [11].

**Table 1:** Assessment of NAFLD Activity Score (NAS).

Group		Steatosis				Lobular Inflammation				Hepatocyte Ballooning			NAS Mean (SD)
		0	1	2	3	0	1	2	3	0	1	2	
	n												
**V (early)**	**6**	**-**	**5**	**1**	**-**	**-**	**2**	**2**	**2**	**-**	**-**	**6**	**5.2 (1.0)**
**GM (early)**	**5**	**3**	**2**	**-**	**-**	**-**	**1**	**3**	**1**	**-**	**-**	**5**	**4.4 (1.1)**
**GR (early)**	**6**	**4**	**2**	**-**	**-**	**-**	**5**	**1**	**-**	**-**	**3**	**3**	**3.0 (0.9)**
**V (late)**	**4**	**1**	**2**	**1**	**-**	**-**	**2**	**2**	**1**	**-**	**-**	**5**	**5.3 (1.3)**
**GM (late)**	**5**	**1**	**2**	**2**	**-**	**-**	**2**	**2**	**1**	**-**	**-**	**5**	**5.0 (1.4)**
**GR (late)**	**6**	**3**	**3**	**-**	**-**	**-**	**3**	**2**	**1**	**-**	**3**	**3**	**3.7 (1.4)**

Figure 1. Tabulation of scoring of steatosis, lobular inflammation, hepatocyte ballooning, and NAFLD activity score in liver sections performed as described in Methods on liver sections obtained from animal livers from the experiment described in

**Figure 3:**
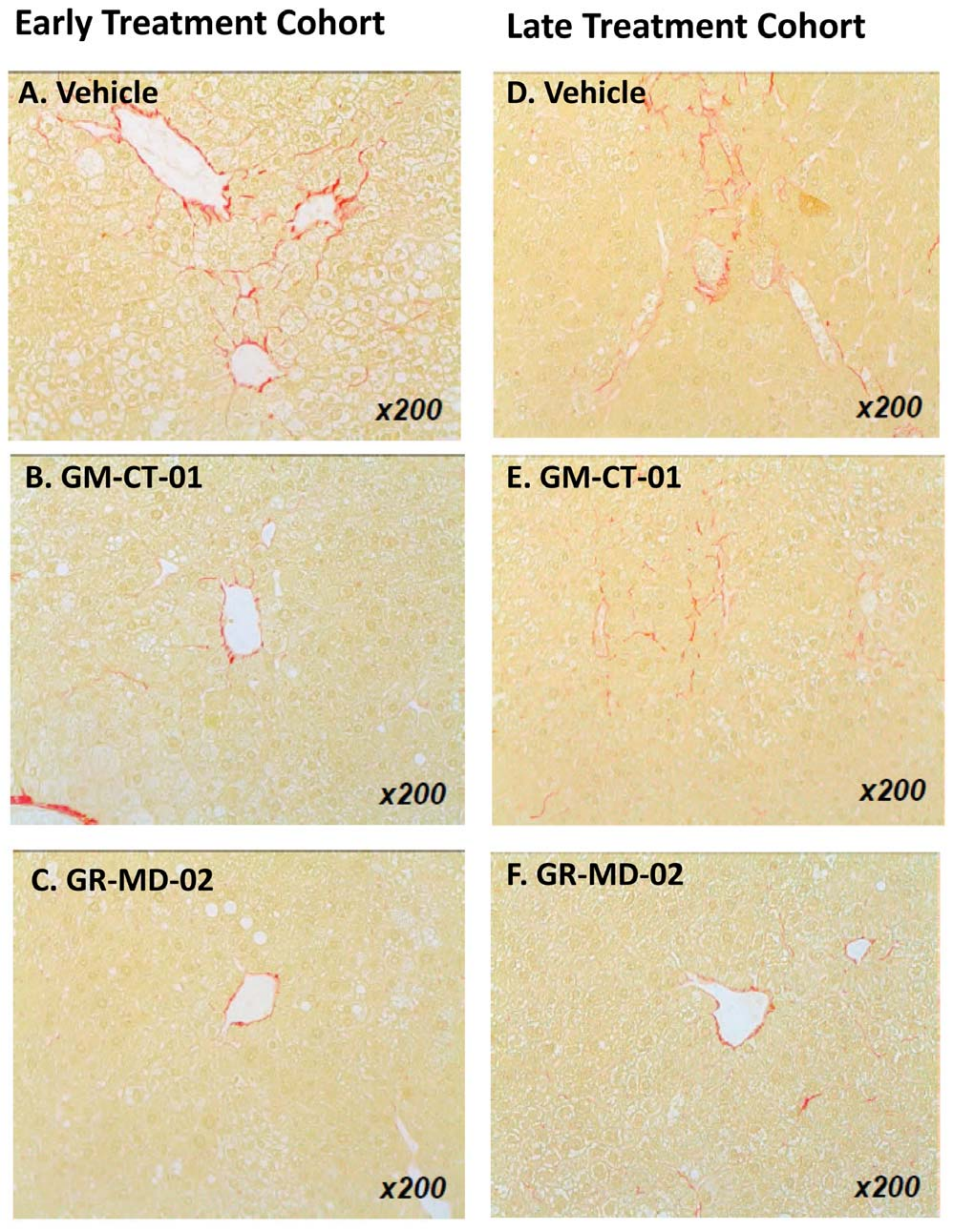
Liver histology stained with Sirius red. Early and late treatment cohorts were administered intravenously twice weekly vehicle (0.9% NaCl), GM-CT-01 (120 mg/kg), or GR-MD-02 (60 mg/kg).

Collagen deposition in the vehicle treated groups was observed mainly in peri-central regions of the liver lobule (Figures 3A and 3D). In the GM-CT-01 group, the collagen deposition tended to decrease compared with the vehicle group (Figures 3B and 3E). In the GR-MD-02 group, the collagen deposition markedly decreased in the peri-central region of the liver lobule compared with the vehicle group (Figures 3C and 3F). Quantification of the area of Sirius red staining showed that the percentage positive area tended to decrease in the GM-CT-01 group and was significantly decreased in both GR-MD-02 early and late treatment groups (Figures 2C and 2D). Therefore, in addition to a reduction in the NAFLD activity score, treatment with GR-MD-02 markedly reduced the deposition of collagen in both the early and late treatment cohorts.

### Dose response of treatment effect

Because of the more robust effect of treatment with GR-MD-02, the effect of dose response on the treatment effect was evaluated. In addition, while the original experiments were conducted with twice weekly dosing, this next set of experiments was done using once weekly intravenous injections, a regimen that would be more likely acceptable in a clinical situation. Groups of 12 mice each were intravenously administered vehicle or GR-MD-02 in doses of 120, 60, 30, or 10 mg/kg once weekly from 6 to 12 weeks of age. In addition, twelve normal mice were included for comparison. Two mice died during the course of the experiment, one in the vehicle group and one in the GR-MD-02 30 mg/kg group, and were therefore not included in the final analysis.

Mean body weight, mean liver weight, liver-to-body weight ratio, mean spleen weight, and spleen-to-body weight ratio at sacrifice were significantly different in the vehicle group compared with the normal group, but there were no significant differences among treatment groups when compared to vehicle (Table 2). Similarly, blood glucose levels significantly increased in the vehicle group compared with the normal group, but there was no significant difference between the vehicle and treatment groups (Table 2).

**Table 2:** Comparison of physical and lab parameters between animal groups in dose response experiment with GR-MD-02 (GR).

Parameter: mean (SD)	Normal	Vehicle	GR 120 mg/kg	GR 60 mg/kg	GR 30 mg/kg	GR 10 mg/kg
	(n = 12)	(n = 11)	(n = 12)	(n = 12)	(n = 11)	(n = 12)
**Body Weight (g)**	**27.1 (1.4)**	23.6 (1.6) **	**23.8 (1.7)**	**23.1 (1.5)**	**21.8 (3.3)**	**22.0 (3.0)**
**Liver Weight (g)**	**1175 (111)**	1955 (412) **	**2035 (144)**	**1821 (147)**	**1782 (252)**	**1709 (207)**
**Liver-Body Weight (%)**	**4.3 (0.4)**	8.2 (1.3) **	**8.6 (0.8)**	**7.9 (0.8)**	**8.3 (1.0)**	**7.8 (0.9)**
**Spleen Weight**	**80 (15)**	226 (99) **	**266 (75)**	**257 (120)**	**262 (63)**	**254 (85)**
**Spleen-Body Weight (%)**	**0.3 (0.1)**	1.0 (0.4) **	**1.1 (0.3)**	**1.2 (0.6)**	**1.2 (0.3)**	**1.2 (0.5)**
**Blood Glucose (mg/dL)**	**147 (42)**	644 (75) *	**691 (88)**	**708 (71)**	**743 (95)**	**717 (113)**
**Plasma AST (U/L)**	**117 (96)**	**154 (55)**	**164 (67)**	**144 (80)**	**133 (66)**	**142 (44)**
**Plasma ALT (U/L)**	**32 (12)**	67 (29) **	**80 (47)**	**73 (59)**	**55 (29)**	**50 (11)**
**Plasma TG (mg/dL)**	**91 (16)**	237 (190) **	**277 (221)**	**211 (109)**	**511 (739)**	**543 (642)**

* p<0.05; ** p<0.01. Treatment groups were compared to Vehicle group using one way ANOVA and bonferoni multiple comparison tests; no differences were found. Normal and Vehicle groups were compared by student's t-test;

Plasma ALT levels were significantly increased in the vehicle group compared with the normal group and tended to decrease with GR-MD-02 treatment, but the differences were not significant (Table 2). Plasma AST levels tended to increase in the vehicle group compared with the normal group, but there were no significant differences between treatment groups (Table 2). Plasma TG levels significantly increased in the vehicle group compared with the normal group, but there were no significant differences in plasma TG levels between the vehicle group and any of GR-MD-02 treatment groups (Table 2).

**Figure 4:**
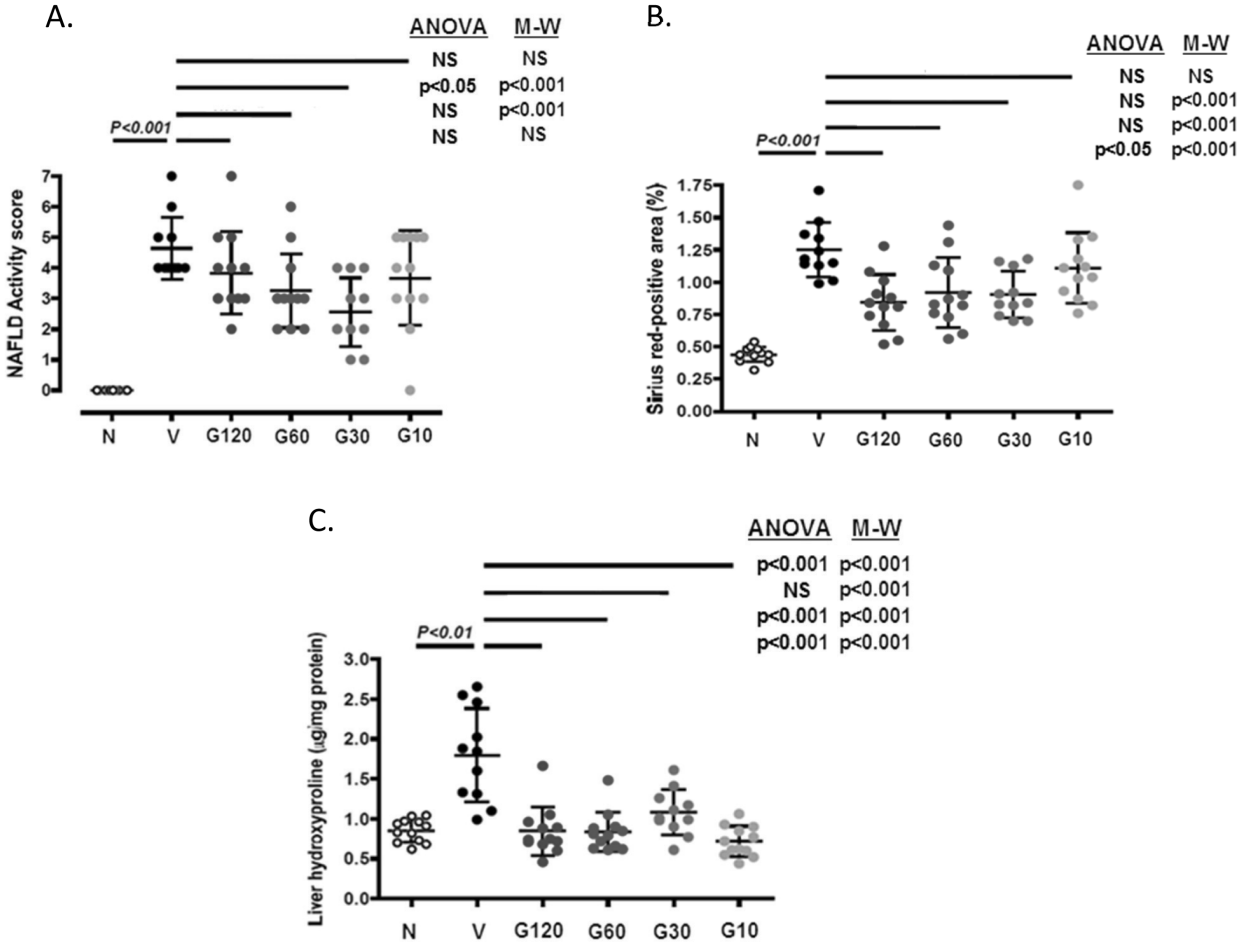
Dose response analysis of GR-MD-02. N  =  normal mice. Treatments were with vehicle (V, 0.9% NaCl) and various doses of GR-MD-02, G120 (120 mg/kg), G60 (60 mg/kg), G30 (30 mg/kg), and G10 (10 mg/kg) each administered intravenously once weekly for six weeks from week 6–12. **A**: NAFLD Activity Score; **B**: Sirius red-positive area; **C**. Liver hydroxyproline content. Differences between normal and vehicle groups were assessed by student t tests. Differences between the vehicle group and the treatment groups were assessed by one way ANOVA followed by Tukey's Multiple Comparison Test and the Mann-Whitney U test (shown in parentheses). *P* values <0.05 were considered significant and the results are expressed as mean ± SD.

The NAFLD activity score was significantly elevated in the vehicle group compared with the normal group (Figure 4A). The blinded histopathologist reported that all GR-MD-02 treatment groups showed obvious reductions of hepatocellular ballooning with marked improvement in lobular inflammation and hepatocellular ballooning observed in the 60 mg/kg and 30 mg/kg groups. These observations correlated with a statistically significant reduction in the NAS of the 60 mg/kg and 30 mg/kg groups when compared with the vehicle group (Figure 4A). The NAS tended to decrease in the GR-MD-02 120 mg/kg and 10 mg/kg groups compared with the vehicle group, but the differences did not reach significance. The lack of dose dependence in the 120 mg/kg group appeared to be related primarily to the fact that inflammation was not improved. Since 120 mg/kg was twice the single dose used in any of the other experiments, this suggests that there may be a therapeutic window of the effect on inflammation.

The Sirius red-positive area significantly increased in the vehicle group compared with the normal group (Figure 4B). Compared with the vehicle group, collagen deposition was significantly reduced in the 120, 60, and 30 mg/kg GR-MD-02 treatment groups and there was a non-significant trend to decreased levels in the 10 mg/kg group (Figure 4B). Liver hydroxyproline was significantly increased in the vehicle group compared with the normal group and was significantly decreased in the all GR-MD-02 groups (Figure 4C) supporting the quantitative collagen assessment. The difference in the results of Sirius red staining and hydroxyproline content in the 10 mg/kg group may be related to differences in the sensitivity and reproducibility of the two the methods.

### Route of administration on treatment efficacy

**Figure 5:**
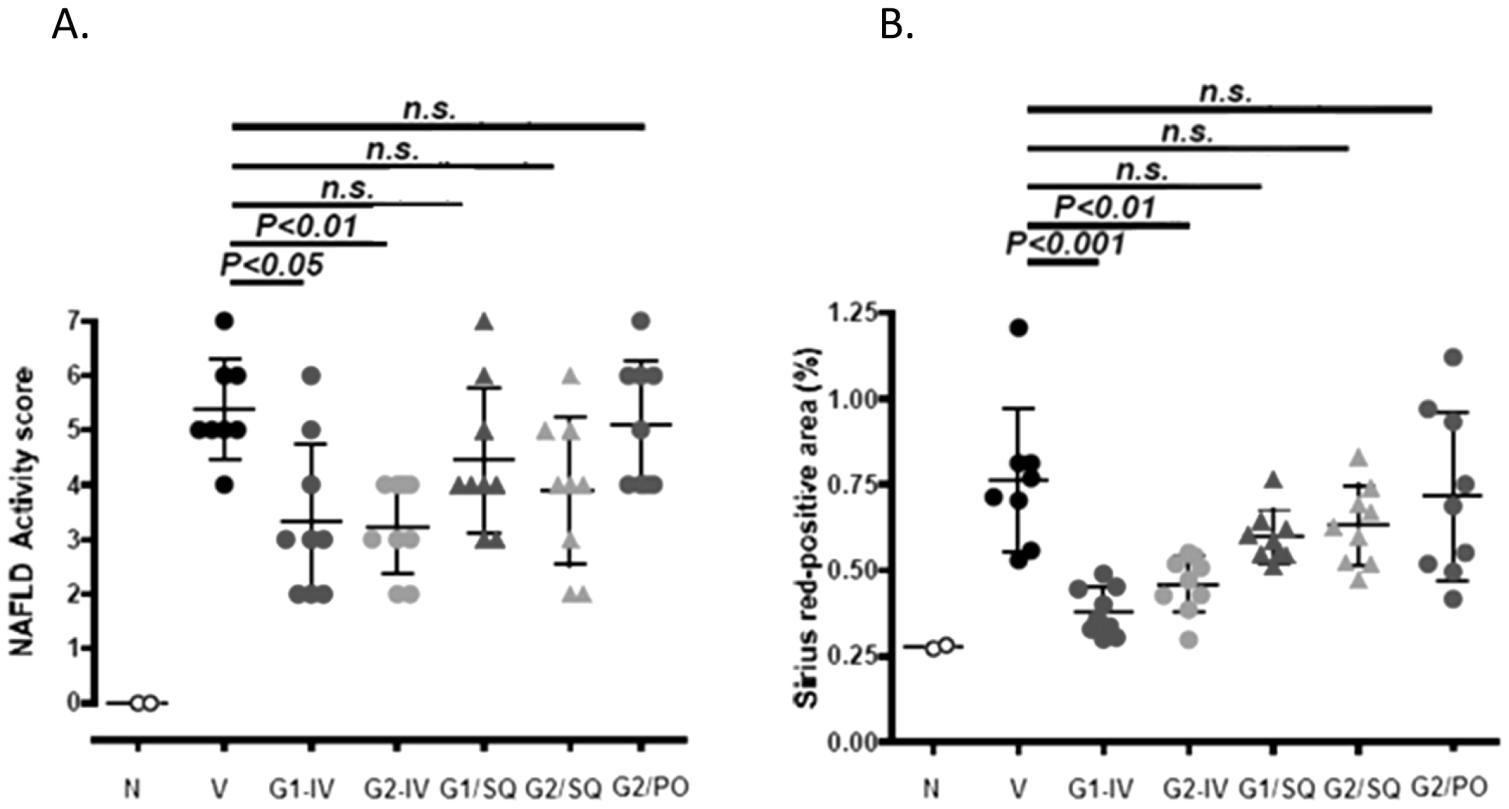
Evaluation of route of administration of GR-MD-02. N: normal mice. Treatments included vehicle (V) (0.9% NaCl three times weekly from weeks 6–9), and intravenous doses of two preparations of GR-MD-02 (40 mg/kg three times weekly from weeks 6–9) sterilized by different methods given either intravenously (G1/IV and G2/IV) or subcutaneously (G1/SQ and G2/SQ). Additionally GR-MD-02 was given orally in a dose of 1500 mg/kg daily (G2/PO). **A**: NAFLD Activity Score; **B**: Sirius red-positive area. Differences between the vehicle group and the treatment groups were assessed by one way ANOVA followed by Tukey's Multiple Comparison Test and the Mann-Whitney U test (shown in parentheses). *P* values <0.05 were considered significant and results expressed as mean ± SD.

Experiments were performed to evaluate the effect of the route of administration of GR-MD-02 (Figure 5). The number of animals evaluated in this study was 8 in the vehicle-treated animals and 9 in each of the treatment groups; 2 normal littermates that did not receive streptozotocin or high fat diet were also evaluated. Similar to previous experiments, the NAS and the percent Sirius red area was markedly increased over normal animals. Treatment with two different preparations of intravenous GR-MD-02, one research grade (G1) and the second GMP grade material (G2), significantly reduced both NAS (Figure 5A) and percent Sirius red area (Figure 5B) when compared to the vehicle group. Subcutaneous administration of GR-MD-02 tended to have decreased activity score and collagen content, but the differences did not reach significance. This correlates with information that serum levels of GR-MD-02 after subcutaneous administration are only approximately 2% of levels obtained with intravenous administration (data not shown). A large daily oral dose of GR-MD-02 had no effect on activity score or collagen content.

### Treatment effect on tissue gal-3

**Figure 6:**
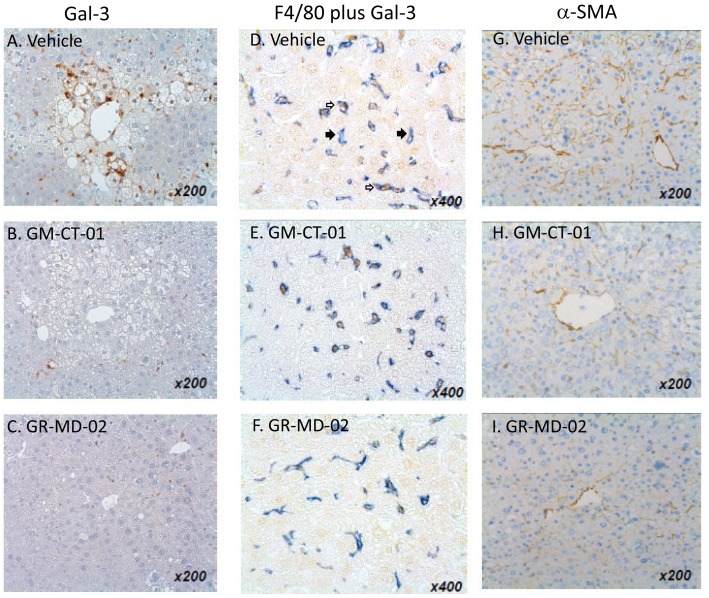
Liver immunohistochemistry. **A–C**: Gal-3; **D–F**: F4/80 plus Gal-3; **G–I**: α-SMA. Immunohistochemistry was performed on liver sections from the late treatment cohort was treated with vehicle (0.9% NaCl, A, D, and G), GM-CT-01 (120 mg/kg, B, E, and H), and GR-MD-02 (60 mg/kg, C, F, and I) each administered intravenously twice weekly. Closed arrows indicated cells that stain only with F4/80 antibody (blue) and open arrows indicate cells that stain with F4/80 antibody (blue) and galectin-3 antibody (brown).

**Figure 7:**
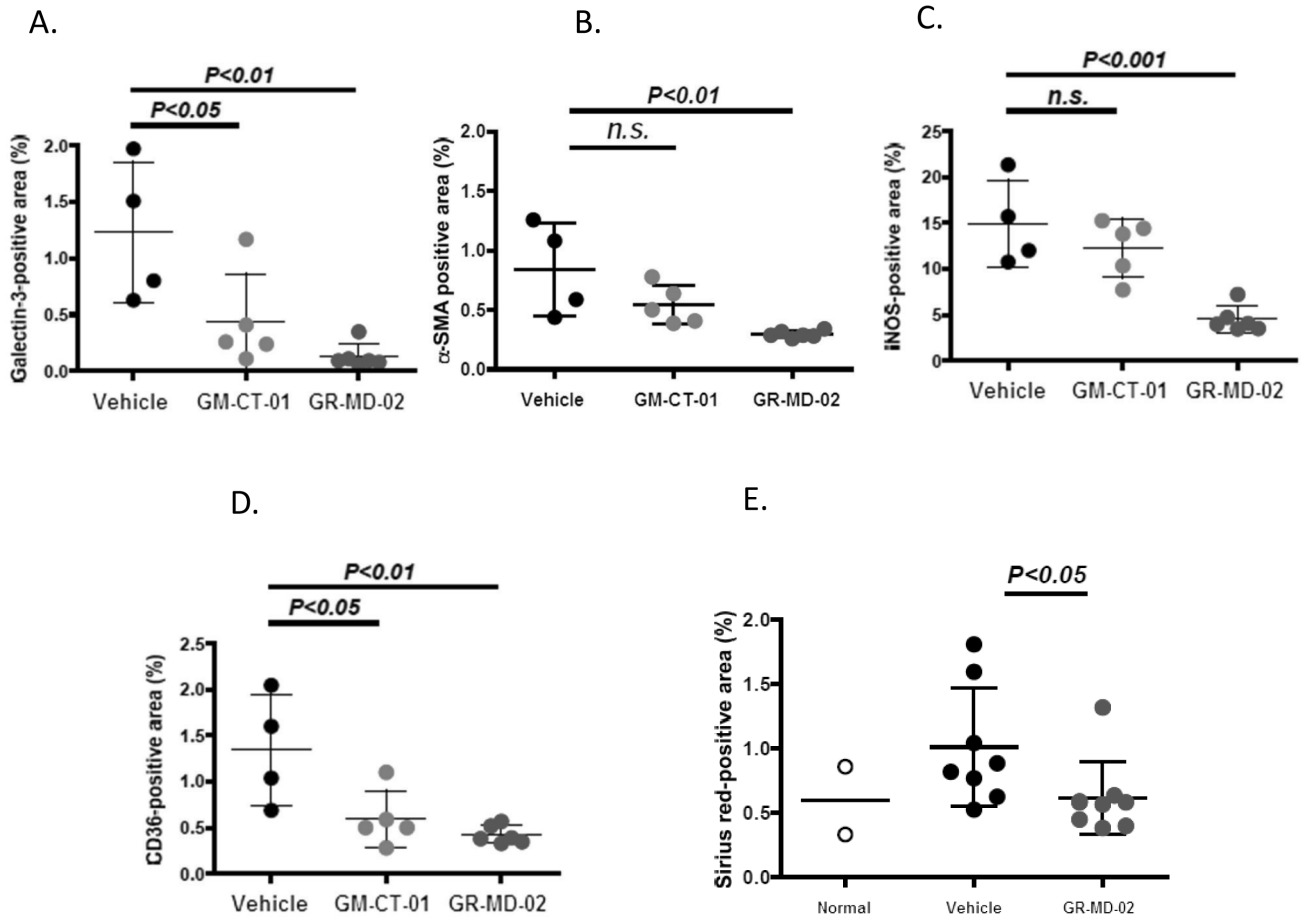
Quantitative analysis of immunohistochemistry and histology. Experiments from Figure 6 and 8. **A**: Gal-3 positive area; **B**: α-SMA positive area; **C**: CD36 positive area; **D**: iNOS positive area; **E**: Sirius red positive area of kidney sections from experiment in figure 8. Differences between the vehicle group and the treatment groups were assessed by one way ANOVA followed by Bonferroni Multiple *P* values <0.05 were considered significant and results expressed as mean ± SD.

In normal liver, there was very low expression of gal-3 with staining observed in scattered macrophage-like cells in the liver lobule with no staining in bile ducts or hepatocytes, as previously shown in mice [18] and reported previously by our group in rat liver [10]. In NASH mice in the late treatment cohort treated with vehicle, we observed increased numbers of gal-3-positive macrophage-like cells in the entire liver lobule, especially in the proximity of hepatocytes with fat deposition and ballooning hepatocytes (Figure 6A). In the treatment groups of this experiment, it was noted by the blinded histopathologist that the number and size of Gal-3-positive cells in the liver lobule were decreased compared with the vehicle (Figures 6B and 6C, Figure 7A). Moreover, the gal-3 positive cells appeared morphologically to be macrophages and were often found clustered around fat-laden and ballooning hepatocytes in the centri-lobular area. The number and the size of F4/80+ cells, a marker of macrophages, in the treatment groups did not change compared with the vehicle group. Double immunohistochemistry demonstrated the co-expression of gal-3 and F4/80. Virtually all of the gal-3-positive cells in the liver of NASH mice were macrophages, whereas there were many macrophages that did not express gal-3 (Figures 6D, 6E, and 6F).

Also in the late treatment cohort of the same experiment, Gal-3 mRNA expression tended to be down-regulated in the treatment groups compared with the vehicle group, although the levels did not reach statistical significance (vehicle: 1.00±0.70, GM-CT-01: 0.77±0.54, GR-MD-02: 0.48±0.13). In contrast, there was no tendency for differences in gal-1 mRNA expression levels between the vehicle group and either of the treatment groups (vehicle: 1.00±0.20, GM-CT-01: 0.95±0.16, GR-MD-02: 1.04±0.13).

Gal-3 immunohistochemistry was also performed on the groups in the dose response experiment. There was a statistically significant increase in the gal-3 positive area in the vehicle group (1.21 (0.74)%) when compare to normal animals (0.11 (0.08)%), showing induction of gal-3 with NASH. The GR-MD-02 60 mg/kg group reached statistical reduction in gal-3 staining (0.65 (0.28)%), while there was less of reduction in other treatment groups with a trend towards lower gal-3 levels in the other treatment groups. These data show that there can be a therapeutic effect on NASH and collagen deposition without a marked reduction in gal-3 expression in macrophages in the tissues.

#### Treatment reduces activated stellate cells

Activated stellate cells, characterized by the expression of α-smooth muscle actin (α-SMA), have been shown to be the primary cell that lays down collagen in fibrogenic liver disease [19]. There was an increase in α-SMA positive cells in the liver lobule of vehicle-treated animals (Figure 6G). The percentage of α-SMA-positive area tended to decrease in the GM-CT-01 group and significantly decreased in the GR-MD-02 group compared with the vehicle group (Figures 6H and 6I and Figure 7B).

#### Treatment reduces iNOS

**Figure 8:**
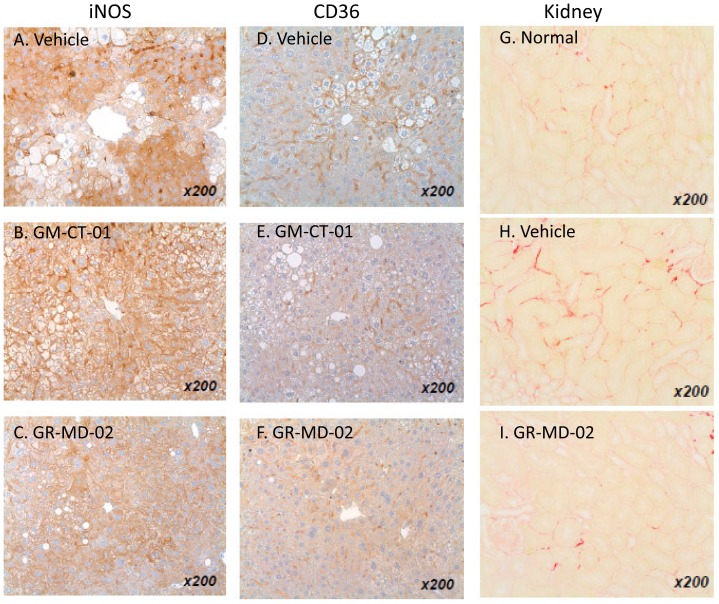
Liver immunohistochemistry and kidney collagen staining. Immunohistochemistry on animals as described in Figure 6. A–C: iNOS; D–F: CD-36; G–I: kidney sections stained with Sirius red from normal animals or animals treated with vehicle or GR-MD-02 (40 mg/kg three times weekly intravenously from weeks 6–9).

The expression of inducible nitric oxide synthase (iNOS) was evaluated as a TH1 inflammatory marker that has been shown to be increased in NASH liver tissue. Positive iNOS staining in the vehicle group was observed in sinusoidal cells and in the cytoplasm of hepatocytes (Figure 8A). The percentage of iNOS-positive area tended to decrease in the GM-CT-01 group and the percentage of iNOS-positive area significantly decreased in the GR-MD-02 group compared with the vehicle group (Figures 8B and 8C and Figure 7C).

#### Treatment reduces CD36

CD36 is an integral membrane glycoprotein found on the surface of many cell types which is a member of the class B scavenger receptor family that binds many ligands including modified low-density lipoprotein (LDL) by oxidation or acetylation and has been shown to be involved in pathogenesis of NASH [11], [20]–[26]. In the treatment groups, the number and size of CD36-positive cells and positive area were decreased compared with the vehicle group (Figure 8D, 8E, and 8F and Figure 7D).

#### Treatment effects on kidney

We also examined the effect of treatment on the kidney, because of the suggestion in the literature that there is a possibility that there could be an increase in kidney damage in NASH with inhibition of gal-3 [11], [27]. Consistent with diabetic nephropathy, kidney sections from the vehicle group exhibited diffuse mesangial expansion and focal mesangial cell proliferation, which were rarely observed in sections from the normal group. In the GR-MD-02 group, mesangial expansion was less frequent and less severe compared with that in the vehicle group (data not shown). Kidney sections from the vehicle group showed collagen deposition in the interstitial region which was increased over normal animals (Figure 8G and 8H). In contrast, the percentage of fibrosis area was significantly decreased in the GR-MD-02 group in comparison to the vehicle group (Figure 8I and 7E). Thus, treatment seemed to improve both glomerulopathy and interstitial fibrosis, suggesting that treatment with this agent gives a different effect than seen in gal-3 null mice.

## Discussion

These experiments show that intravenous administration of the galectin-binding drug GR-MD-02 had reproducible efficacious effects in a murine model of steatohepatitis. Treatment reduced the NAFLD activity score which sums the characteristic histological findings of steatohepatitis including steatosis, hepatocyte ballooning, and inflammatory infiltrate. Moreover, treatment prevented accumulation of collagen and/or reduced accumulated collagen in the liver. The efficacy of treatment was unrelated to NASH progression as the positive effects were seen whether the drug was started early or late in the pathological process. The results of treatment efficacy were consistent between three separate experiments with different designs and dosing regimens.

These results are consistent with the resistance of gal-3 null mice to the development of NASH and fibrosis when fed a high fat diet [11]. However, as outlined in the Introduction, other investigators have found the opposite effect in gal-3 null mice with NASH in aging mice or induced by the CDAA diet. While there is no obvious explanation for the different findings of these two groups, the preponderance of data from other gal-3 null mouse studies on fibrosis in liver [7], kidney [9], [28], lung [8], and heart [29] demonstrates that gal-3 appears to be integral to accumulation of fibrosis in parenchymal tissue. In addition, while the use of gal-3 null mice is a powerful biological model, there are some drawbacks including the obvious potential impact of background mouse strain. Also, both intracellular and extracellular gal-3 is eliminated in the null mice. The extracellular effects of galectins are related to their lectin properties to bind to glycoproteins whereas their intracellular effects are related to protein-protein interactions [30]. Treatment with GR-MD-02, a complex carbohydrate with galactose residues would be expected to interfere with lectin effects predominantly on the cell surface and in the extracellular space.

While the degree of collagen deposition in the model of NASH used in these studies is modest, as in many animal models of NASH, we have previously reported evidence that the same drug agents are effective in reducing much greater degrees of fibrosis and cirrhosis in thioacetamide-treated rats [10]. In these previous studies, the regression in cirrhosis was associated with a reduction in portal hypertension, demonstrating that the change in liver architecture has a physiological effect on liver blood flow and/or resistance. Therefore, it appears that these drugs have effects on the early pathophysiological collagen deposition in NASH, including peri-central and peri-sinusoidal deposition of collagen and potentially later stages of fibrosis and cirrhosis.

The presumed proximate mechanism of action of the drugs used in this study is related to gal-3 binding. Gal-3 has a carbohydrate recognition domain (CRD) which is shared among galectin proteins [31], but in contrast to other galectin proteins, it has a long N-terminal domain that is involved in forming multimers [32]. Gal-3 binds poorly to single galactose molecules [33], more avidly to galactose containing disaccharides [34], and most avidly to larger molecules such as glycoproteins with galactose residues [33]. We have shown that our carbohydrate drugs bind to the gal-3 CRD through somewhat different sets of amino acid residues and the affinity at 50% saturation of GR-MD-02 and GM-CT-01 to gal-3 is 2.9 µM and 2.8 µM, respectively ([10] and unpublished data). This compares to gal-1 binding affinities for GR-MD-02 and GM-CT-01 of 8 µM and 10 µM, respectively [35]–[37]. Although galectins are defined by their ability to bind to model carbohydrates containing galactose, such as N-acetyllactosamine, individual galectins appear to bind to different sets of glycans on glycoproteins, thus providing specificity between galectins [38]. For example, galectin-1 and galectin-3 bind to distinct cell surface receptors on T-cells [39]. There are many reported potential ligands for the lectin properties of galectin-3 including laminin, integrins, collagens, fibronectin, elastin, mucins, CD4+, CD8+, TGFBR, neural cell adhesion molecules, and many others [40]. Binding of galectin-3 to N-glycans has been connected to multiple cellular processes including cell adhesion and migration, immune cell function, inflammation, and neoplasia [31], [41]–[45]. It is likely, that inhibition of galectin-3 modulates multiple protein interactions in the extracellular space thereby altering cellular function.

We have not determined in these studies which gal-3 protein interactions are abrogated by drug treatment. However, we have data that suggest some downstream processes that are affected, one of which seems to involve macrophages. Gal-3 is expressed at much higher levels in macrophages than other cell types [46] and is important for macrophage function in fibrotic disease [9], [28], [47], including regulation of alternative activation of macrophages [28]. Macrophages are pivotal to the development and resolution of collagen deposition in organs [48] and are clearly important in liver fibrosis [49]. Moreover, it is now clear that activated macrophages differentiate into a number of different subtypes which have distinct functions along the continuum from inflammation and fibrogenesis to resolution of fibrosis. The classically activated M1-macrophages have an acute inflammatory phenotype, are aggressively phagocytic for bacteria, and produce large amounts of cytokines. The alternatively activated, anti-inflammatory M2-macrophages can be separated into three subgroups that have different function in immune regulation, tolerance, and tissue repair or wound healing. Recently, a new subtype of M2-macrophages was identified that is critical for resolution of fibrosis in the liver [50].

The improvement we found in the activity of NASH and reduction in fibrosis was associated with reduction in the expression of gal-3 protein, although it was not necessary to have a marked reduction to observe the therapeutic effect. Gal-3 was expressed predominantly in a subset of macrophages that appear in areas of greatest hepatocellular damage. The reduction in immunohistochemical staining of gal-3 was not related to masking epitopes due to drug binding or exclusively enhanced degradation of the protein since gal-3 mRNA was also reduced. It is not clear whether the reduction in gal-3 in macrophages is due to a primary effect of drug binding to gal-3 or rather to a shift in macrophage phenotype that expresses lower levels of gal-3. Our studies also suggest another potential role of these complex carbohydrate drugs on macrophages since we observed that the drugs are taken up by macrophage phagocytosis (data not shown). Gal-3 is important for macrophage phagocytosis [51] and phagocytosis appears to be important in macrophage polarization, in particular with the activation of a restorative/reparative M2 phenotype [50]. While these results suggest that the macrophage, a well described key cell in fibrogenesis, may be an important target for these complex carbohydrate drugs, additional investigations will be required to elucidate the mechanisms.

Our results show that expression of iNOS, a TH1 marker of inflammation, is markedly reduced in NASH livers following GR-MD-02 treatment. The expression of iNOS has been shown to be increased in NASH animal models [52] and human disease [53], [54] and is an important downstream target of multiple cytokines in complex inflammatory processes [55]. While the proximate molecular pathway for reducing iNOS is not known, it appears that the effect of GR-MD-02 on the inflammatory process, possibly due to an effect on macrophages, results in decreased iNOS which may have an important effect on liver inflammation and damage. This finding also suggests that the effect of gal-3 and inhibitors on macrophage phenotype and/or function may be complex affecting not only the M2 phenotype, but also the pro-inflammatory M1 phenotype, as shown in liver toxin damage by Dragomir, et al.[56], [57].

The results of the expression of CD36 in our experiments may also have a link to the therapeutic effect. Iacobini, et al [11] have shown that gal-3 is involved in the early steps of NASH pathogenesis including fat accumulation, hepatocyte damage and inflammation. Gal-3 null mice had decreased levels of lipoxidation and glycation products (ALE-advanced lioxidation endproducts; AGE-advanced glycation endproducts) and oxidative ER stress which were associated with a lack of increase in ALE/AGE-receptor expression. An important component of this receptor is CD36 which was not increased in gal-3 null mice fed a high fat diet. They concluded that the prevention of NASH and the loss of receptor function in gal-3 null mice implied that gal-3 is the main scavenger receptor involved in update of ALE/AGE. In our experiments, we show that CD36 expression is markedly reduced in NASH mice treated with GR-MD-02. This suggests that treatment with GR-MD-02 may have a similar effect on scavenger receptor function as seen in the gal-3 null mice. It is important to note that these authors suggested that inhibition of this receptor function might increase circulating ALE/AGE and cause other end organ damage, such as kidney. However, in contrast to this hypothesis, we show that treatment with GR-MD-02 actually improves kidney damage found in this NASH model.

Collagen accumulation is a later step in the pathological process of NASH which has also been shown to be related to gal-3 expression [11]. We show that activated stellate cells, the primary cell type for the synthesis of collagen in liver fibrogenesis [58]–[62], are reduced with GR-MD-02 treatment. This would be expected to result in reduced expression of collagen and reduced accumulation. While it is known that gal-3 is directly involved in stellate cell activation [7], [63], it is uncertain whether the effects seen are related to a direct effect on stellate cells or a secondary effect based on alterations in the cytokine environment. In this regard, we showed minimal effects of these drugs on a stellate cell line, LX-2, in previously reported experiments [10]. An additional effect on collagen accumulation could be related to an alteration in macrophage phenotype which could lead to decreased activation of stellate cells or a direct effect on catabolism of collagen. In any event, the net end effect of drug treatment is a reduced amount of collagen in the liver.

In conclusion, GR-MD-02 and to a lesser extent GM-CT-01, both complex carbohydrates that bind to gal-3, ameliorate the findings of fat accumulation, hepatocellular damage, inflammation and fibrosis in a mouse model of NASH. These effects are associated with a reduction in gal-3 expressing macrophages, reduction in iNOS expression, reduction in components of the ALE/AGE receptor complex, and reduction in activated stellate cells. Taken together, the effect of drug treatment appears to interfere with multiple pathogenic pathways that reduce NASH and fibrosis, effects that have been shown to be related to the absence of gal-3 in null mice. While further experiments are required to elucidate the various mechanisms of the therapeutic effect, the exploration of GR-MD-02 as a treatment for human NASH with fibrosis is a promising approach to this disease where there are no approved therapies.
